# Complete Genome Sequence of *Citrobacter freundii* Myophage Mordin

**DOI:** 10.1128/genomeA.01203-15

**Published:** 2015-10-15

**Authors:** Jingwen Guan, Jeffrey D. Snowden, Jesse L. Cahill, Eric S. Rasche, Gabriel F. Kuty Everett

**Affiliations:** Center for Phage Technology, Texas A&M University, College Station, Texas, USA

## Abstract

*Citrobacter freundii* is a Gram-negative opportunistic pathogen that is associated with urinary tract infections. Bacteriophages infecting *C. freundii* can be used as an effective treatment to fight these infections. Here, we announce the complete genome sequence of the *C. freundii* Felix O1-like myophage Mordin and describe its features.

## GENOME ANNOUNCEMENT

*Citrobacter freundii* is a member of the family *Enterobacteriaceae*. *C. freundii* is a significant cause of opportunistic infections, such as neonatal meningitis, septicemia, and brain abscesses (1). Due to increasing antibiotic resistance among *C. freundii* strains (2), bacteriophages may be an alternative therapy against this pathogen. Here, we present the complete genome sequence of *C. freundii* Felix O1-like myophage Mordin.

Bacteriophage Mordin was isolated from a water sample collected at College Station, TX. Phage DNA was sequenced in an Illumina MiSeq 250-bp paired-end run with a 550-bp insert library at the Genomic Sequencing and Analysis Facility at the University of Texas (Austin, TX). Quality-controlled trimmed reads were assembled to a single contig of circular assembly at 65-fold coverage using SPAdes version 3.5.0 (3). The contig was confirmed to be complete by PCR using primers that face the upstream and downstream ends of the contig. The products from PCR amplification of the junctions of concatemeric molecules were sequenced by Sanger sequencing (Eton Bioscience, San Diego, CA). Genes were predicted using GeneMarkS (4) and corrected using software tools available on the Center for Phage Technology (CPT) Galaxy instance (https://cpt.tamu.edu/galaxy-pub/). Morphology was determined using transmission electron microscopy performed at the Texas A&M University Microscopy and Imaging Center.

Mordin has an 89,596-bp double-stranded DNA (dsDNA) genome containing 138 predicted coding sequences. It has a coding density of 89.3% and a G+C content of 38.8%. The G+C content of Mordin is similar to that of *Salmonella* phage Felix O1 (accession no. NC_005282) (39.0%) (5), but it is significantly lower than that of *C. freundii* (51.61%) (6). The *Salmonella* G+C content (approximately 52%) is similar to that of *Citrobacter* (7). A low G+C content compared to its host seems to be a common feature of Felix O1-like phages (5, 8). Of the 138 predicted coding sequences, 106 are hypothetical novel or conserved genes, and 32 were given a putative function based on BLASTp and InterProScan analyses (9, 10). Sequence analysis using Emboss Stretcher showed that Mordin shares 46.7% and 48.8% nucleotide sequence identity across the genome with Felix O1 and *Escherichia* phage wV8 (accession no. NC_012749), respectively (11). Mordin is syntenic with Felix O1, and despite low sequence identity, 114 of 138 (82.6%) putative coding sequences are similar, according to CoreGenes (12). Most of the differences between the two phages occur in hypothetical proteins of unknown function. Mordin contains 25 tRNA genes, similar to the 22 tRNAs identified in Felix O1 (5). As with Felix O1, Mordin contains *rIIA* and *rIIB* genes and was opened just upstream of the *rIIA* homolog for annotation purposes. Mordin encodes a single HNH homing endonuclease, whereas five have been identified in Felix O1. Interestingly, a 26-bp repeat (consensus, 5′-CCAACAATCCTAAACTGGAGAATCTA-3′), reminiscent of start-associated sequences (SASs) described in cluster K mycobacteriophages, was identified upstream of the translational start of 8 hypothetical conserved/novel genes on the left arm of the genome (13). The role of SASs in gene expression is currently unknown (14).

### Nucleotide sequence accession number.

The genome sequence of phage Mordin was deposited in GenBank under the accession no. KT363872.
